# Improving the confidence and competence of junior doctors in conducting seclusion reviews

**DOI:** 10.1192/bjo.2021.437

**Published:** 2021-06-18

**Authors:** Mostafa Shalaby, Mehtab Rahman

**Affiliations:** Central & North West London NHS Foundation Trust

## Abstract

**Aims:**

- To improve the quality and consistency of medical seclusion reviews at St Charles Hospital and across the Trust.
- To ensure at least 80% compliance with minimum standards for seclusion review documentation by the end of December 2020.
- To increase doctors' mean perceived competence and confidence scores to 4.5/5 by the end of December 2020

**Method:**

Seclusion is commonly used to manage patients at high risk of aggression or violence, but is a high risk and very restrictive intervention. As such, it requires regular nursing and medical reviews. Work has been done recently at St Charles to improve the timeliness and effectiveness of nursing reviews including detailed guidance. Medical reviews are usually performed by junior doctors, many with limited experience in psychiatry. There is

- A lack of consistent local or national guidance for junior doctors undertaking seclusion reviews
- The quality and scope of these reviews is not consistent
- There may be a need to ensure that there is more standardization and to improve junior doctors' confidence – and therefore patient safety and experience – overall.
- The following interventions were used to improve the quality of seclusion reviews at the hospital:
- Minimum standard guidelines
- Presenting in Restrictive interventions meeting.
- Feedback from PICU consultants for guidelines
- Changing guidelines

Future plans:

- Guidelines teaching (Early November)
- Re-audit and new survey (Early November)
- Simulation training (Mid November)
- Seclusion teaching video (Early December- to be ready for Induction)
- Re-audit and new survey (Beginning of April)

**Result:**

Surveys were conducted before and after quality improvement interventions were put in place. The average confidence levels of junior doctors increased from 38.5% to 87% following these interventions.

**Conclusion:**

Revision of seclusion guidelines, junior doctor teaching and simulation training are effective interventions to improve junior doctor confidence levels in conducting seclusion reviews.

